# Text Message Interventions in Adolescent Mental Health and Addiction Services: Scoping Review

**DOI:** 10.2196/16508

**Published:** 2021-01-08

**Authors:** Sarah MacDougall, Susan Jerrott, Sharon Clark, Leslie Anne Campbell, Andrea Murphy, Lori Wozney

**Affiliations:** 1 Maritime SPOR SUPPORT Unit Halifax, NS Canada; 2 Nova Scotia Health Halifax, NS Canada; 3 IWK Health Halifax, NS Canada; 4 Department of Community Health and Epidemiology Dalhousie University Halifax, NS Canada; 5 College of Pharmacy Dalhousie University Halifax, NS Canada; 6 Mental Health and Addictions Policy and Planning Nova Scotia Health Dartmouth, NS Canada

**Keywords:** adolescent, mental health, eHealth, text messaging, SMS, information science, cell phone, implementation, review

## Abstract

**Background:**

The vast majority of adolescent mental health and substance use disorders go undiagnosed and undertreated. SMS text messaging is increasingly used as a method to deliver adolescent health services that promote psychological well-being and aim to protect adolescents from adverse experiences and risk factors critical for their current and future mental health. To date, there has been no comprehensive synthesis of the existing literature on the extent, range, and implementation contexts of these SMS text message interventions.

**Objective:**

The objective of this scoping review was to map and categorize gaps in the current body of peer-reviewed research around the use of SMS text messaging–based interventions for mental health and addiction services among adolescents.

**Methods:**

A scoping review was conducted according to Levac’s adaptation of Arksey and O’Malley’s methodological framework for scoping reviews in six iterative stages. A search strategy was cocreated and adapted for five unique databases. Studies were screened using Covidence software. The PICO (patient, intervention, comparator, outcome) framework and input from multiple stakeholder groups were used to structure and pilot a data extraction codebook. Data were extracted on study methodology and measures, intervention design, and implementation characteristics, as well as policy, practice, and research implications.

**Results:**

We screened 1142 abstracts. Of these, 31 articles published between 2013 and 2020 were eligible for inclusion. Intervention engagement was the most common type of outcome measured (18/31), followed by changes in cognitions (16/31; eg, disease knowledge, self-awareness) and acceptability (16/31). Interventions were typically delivered in less than 12 weeks, and adolescents received 1-3 messages per week. Bidirectional messaging was involved in 65% (20/31) of the studies. Limited descriptions of implementation features (eg, cost, policy implications, technology performance) were reported.

**Conclusions:**

The use of SMS text messaging interventions is a rapidly expanding area of research. However, lack of large-scale controlled trials and theoretically driven intervention designs limits generalizability. Significant gaps in the literature were observed in relation to implementation considerations, cost, clinical workflow, bidirectionality of texting, and level of personalization and tailoring of the interventions. Given the growth of mobile phone–based interventions for this population, a rigorous program of large-scale, well-designed trials is urgently required.

## Introduction

### Limits of Face-to-face Mental Health and Addiction Care for Adolescents

Many mental health disorders emerge in adolescence, which contribute to the existing burden of disease among young people and later in life [1]. More than 50% of adult mental disorders have their onset before the mid-teen years [2,3]. Furthermore, adolescents experiencing depressive symptoms more than two-standard-deviations above the mean predicts a twofold to three-fold greater risk for an adult major depressive episode [4]. Notably, a survey of 10,123 adolescents aged 13 to 18 years in the continental United States showed that 40% of participants with one disorder also met criteria for another lifetime disorder [5]. Substance use and mental health disorders, for example, commonly co‐occur [6] and are closely related to increased morbidity and mortality [7]. A recent meta-analysis of 41 studies conducted between 1985 and 2012 in 27 countries estimated a global point prevalence of mental disorders in children and adolescents of 13% [8]. From a global perspective, neuropsychiatric disorders are the leading cause of years lost due to disability among 10- to 24-year-olds [9].

The persistent lack of available services to identify and meet these needs is concerning. While several face-to-face psychological therapies have demonstrated effectiveness in the treatment of mental health and addictions, many children and adolescents do not receive or have access to these treatments [10,11]. Despite decades of effort to improve access, demand continues to outstrip provider capacity for face-to-face services [12,13]. In particular, children experiencing poverty [14], children in rural areas [15], and youth and families who experience self-stigma due to prejudice and stereotyping [16] consistently face unequal access or barriers to care. Even when face-to-face services are available, treatment engagement is challenging. For example, high levels of missed appointments and premature termination of therapy are significant for adolescents, with no-show and attrition rates of 40%–60% commonly reported for this population [17]. Given this global burden and the unsustainability of traditional intervention approaches to meet the rising demand, it is vital for new lines of research to generate innovative strategies and interventions for adolescent mental health and addictions.

Mental health systems facing these challenges increasingly look to advancements in information and communications technologies to augment provider capacity, promote healthy behavior and lifestyle changes, and overcome barriers that limit help-seeking [18,19]. However, the technology marketplace is continuously evolving into more dynamic, diverse, and sophisticated functionalities. As technologies are expected to increase in scope and impact, rapid updating and analysis of emerging evidence is needed to inform future research and signal new interventions to policy makers in order to accelerate implementation of high-quality services once effectiveness of these interventions has been established.

### Leveraging Trends in Text Messaging for Adolescent Mental Health Research

One rapidly growing field of study is the use of SMS text messaging for delivering mental health and addiction interventions. Seven billion people, or 95% of the global population, live in an area covered by a mobile-cellular network [20], making SMS text messaging one of the most widely used information and communication technologies. Texting is used by most adolescent cell phone owners and has surpassed phone calls, instant messaging, social network messaging, and face-to-face talking as the preferred mode of communication for this age group [21]. Adolescents report convenience, discreetness, increased communication effectiveness, and reduced anxiety associated with texting or talking on the phone in comparison to in-person evaluations with a physician [22] as reasons for preferring this modality. The immediacy of reaching adolescents through texting is apparent, with 91% of texts read within the first 3 minutes of receipt [23]. Cell phone ownership among 12- to 17-year-olds has been steadily rising over the past several years and is consistent across race and gender groups [24]. Reports from 2019 indicated that among US teens, 69% have a smartphone by the age of 12 [25]; however, teens from lower-income families are slightly less likely to own cell phones than teens from higher-earning families [24]. Although smartphone ownership is rapidly growing, only about a third of the world’s population (approximately 2.6 billion) used a smartphone in 2017 [26], compared to over 5 billion mobile phone subscribers. Even using conservative estimates, simple SMS text messaging that does not require smartphone capabilities will remain an important tool to reach adolescents for some time to come. Consequently, researchers and decision makers need strategic guidance on where new investments in SMS text messaging intervention development and testing are best positioned in the future.

### Related Work and the Need for Mapping the Current Evidence Base

Texting is among the most frequently used technologies for low-intensity behavioral health interventions [27] and has demonstrated effectiveness in supporting a range of healthy behavior changes among adolescents with diabetes [28] and obesity [29] and for many other topics including sexual health [30] and contraception [31]. Interventions in these other health domains typically make use of multiple persuasive system design features (eg, personalization, reminders, feedback, branching/tailoring) [32], but there are significant gaps in the knowledge base about breadth of features used to deliver service through this modality and the contexts and populations in which SMS text messaging interventions focused on mental health or addictions have been tested.

The most recent synthesis of SMS text messaging intervention research for mental health or addictions present several limitations for addressing adolescent population needs. A 2016 review of SMS text messaging mental health interventions by Watson et al [33] excluded studies involving children and adolescents, and reviews by Berrouiguet et al [34] and Rathbone and Prescott [35] did not provide information on the target age range of studies in their review, making it challenging to conduct any subanalysis of adolescent interventions specifically. Importantly, findings from these adult-focused reviews may not be generalizable to adolescent populations due to the unique developmental changes adolescents experience and their different technology preferences. A 2014 meta-analysis of 14 adolescent-focused intervention studies using SMS text messaging revealed a summary effect size of 0.25 on measures of substance use reduction [36]. However, 11 of the studies focused on tobacco use alone, making the generalization to the broader mental health and addictions continuum limited. A review by Badawy and Kuhns [37] of mobile phone interventions targeting adolescents published between 1995 and 2015 limited inclusion to studies where the primary or secondary outcome related to preventive behavior adherence, meaning that the review provides only a partial synthesis of SMS text messaging research across the full continuum of care. The most recently published relevant review was by Garrido et al in 2019 [38] and synthesized data on a broad range of mobile phone interventions for adolescent anxiety and depression, identifying only 4 text messaging interventions of any study design in their search strategy. Cursory scans of recently published literature suggest there are significantly more primary studies on mental health and addiction interventions for adolescents using text messages than have been previously reviewed. Further, limited discussion in previous reviews on the implementation characteristics of interventions under study (eg, costs, information and communications technology infrastructure required, provider training requirements) provide decision makers with few insights to inform real-world requirements of offering these interventions as sustainable services.

It is clear from the gaps identified that the foundational understanding of how these interventions have been implemented and designed, the measures used to evaluate impact, the contexts and mechanisms identified by researchers to explain intervention impacts, and recommendations being offered for policy, practice, and future research requires updating. The objective of this scoping review was to understand the current state of peer-reviewed research around the use of text message–based interventions for mental health and addiction services among adolescents.

Specifically, the review proposed to answer the following questions: What outcomes are measured to determine effectiveness and engagement? What are the technological and clinical design features of these interventions and services? What implementation contexts, mechanisms, barriers, and facilitators are described? What are the recommendations for practice, policy, and research reported by study authors?

## Methods

### Overview

A scoping review design is ideal for broad mapping and characterizing of existing research [39]. Scoping reviews share a similar process to systematic reviews, since they both are rigorous and transparent in identifying eligible literature but are divergent in purpose. Using a scoping review framework allows for broad exploration of the research to map key concepts, evidence types, and gaps in research in a defined field [40].

The Levac adaptation to Arksey and O’Malley’s [41] methodological framework for scoping reviews was applied in six iterative stages: (1) identifying the research question, (2) identifying relevant studies, (3) selecting studies, (4) charting the data, (5) collating, summarizing, and reporting on the articles and (6) consulting with stakeholders. The PRISMA (Preferred Reporting Items for Systematic Reviews and Meta-Analyses) guideline extension for minimum reporting standards in scoping reviews [42] and Joanna Briggs Institute recommendations for scoping reviews [43] were also followed.

### Search Strategy

The search strategy was developed in collaboration with an evidence synthesis specialist at the Maritime SPOR SUPPORT Unit (MSSU), which is an organization that provides support to researchers and brings together key stakeholders to work on government priority projects. MEDLINE, Embase, PsycINFO, CINAHL, and Scopus databases were searched to identify a broad range of articles in October 2018. A search conducted in the Ovid MEDLINE “In-Process & Other Non-Indexed Citations” database was used to reduce the chance of omitting articles not included in PubMed and to capture the most recent literature possible. An example of the search terms and search strategy is available in Multimedia Appendix 1. Reference lists of related reviews were hand-searched for any additional citations. The search was updated by an evidence synthesis specialist at the MSSU on June 1, 2020.

### Study Selection

After removing duplicates, titles and abstracts were uploaded into Covidence software (Covidence). The extensive time and effort requirements for conducting an unfunded review [44] and stakeholders on the overarching project team promoting rapid review approaches to inform decision making shaped the review protocol. To expedite the process while maintaining rigor, the primary author, with experience in eMental Health–related systematic reviews and text messaging intervention design (LW), completed screening at the title and abstract level, following defined eligibility criteria (see Multimedia Appendix 2). Any abstracts considered questionable were moved to the full-text review phase. For titles and abstracts identified in our updated search in June 2020, two reviewers (LW and SM) independently screened titles and abstracts. No test of agreement between reviewers was conducted; instead, all discrepancies were discussed until a decision on moving to full-text review was made.

### Eligibility Criteria

The screener followed the “excluded terms” approach outlined by Carter [45] to improve accuracy and efficiency of single-screener processes without compromising the quality. To this end, several “exclude” terms were generated to quickly reject studies at the title/abstract level that had a high likelihood of being excluded. Exclude terms included HIV, neurodevelopmental, domestic violence, infant, pregnancy, cancer, sexual health, and contraception.

As several meta-analyses and systematic reviews of tobacco and smoking cessation interventions, including a 2016 smoking cessation Cochrane review [46], have already established high-quality evidence for SMS text messaging interventions in that area, intervention studies that focused only on smoking cessation were excluded in order to focus on mapping and characterizing the less well-established literature.

The inclusion criteria were as follows: the article was a primary study or abstract reporting outcome data; the study targeted children or adolescents (included in the age range ≤18 years) by design; text messages were one of the primary delivery mechanisms; the study was published in English; and the intervention was for mental health and addiction care (eg, symptom management, appointment reminders).

The exclusion criteria were as follows: the article was a systematic review, commentary, editorial, or protocol; the study targeted parents of children and adolescents receiving mental health care; the study targeted only smoking cessation or smoking prevention; SMS text messaging was only used for research data collection and had no therapeutic/educational purpose; or the intervention targeted a subgroup of patients with mental health as a secondary issue relative to an overarching medical condition (eg, cancer, HIV, pregnancy).

Full-text review was completed independently by one reviewer (Swati Rathore) at the outset. A validity check on a randomly selected set of 20 full texts was undertaken independently by a second reviewer (SM) to explore potential bias or inconsistency. Excellent interrater agreement (κ=0.90) was established. Questionable studies were discussed and resolved through consensus with a third reviewer (LW). In the case of a study that had a published protocol as well as a study outcome paper, only the outcome paper was included.

### Data Extraction

Suggestions on synthesizing evidence from complex interventions were followed [47]. Briefly, such frameworks emphasize that decision makers are more interested in knowing when the intervention works (delivered by whom, how, how often, and in which setting) than in answering the simple question of “does it work?”. A codebook was drafted (LW and Swati Rathore) based on the PICO (patient, intervention, comparator, outcome) framework. Through consultation and discussion with a range of stakeholders (psychologists/clinicians, academic researchers, patient advisors, policy and planning experts, and mental health system administrators at the local and provincial level), the codebook was refined to highlight implementation and context features identified as “high value” information for decision makers. The data extraction form included the following 7 key sections: (1) *study identification* (ie, citation, year of publication, publication type); (2) *outcomes* (ie, purpose of the service or intervention, outcome types [health system, intervention acceptability, intervention engagement, clinical, physical, cognitive, emotional, functioning and coping, relationships, relaxation skills, compliance and adherence, behavioral], validity and reliability of outcome measure, name of measure, main findings, covariates); (3) *evaluation methods* (ie, sample size of the intervention group, study design, reporting of adverse events or safety issues, baseline data collection, follow-up schedule); (4) *intervention design* (ie, name of intervention, quantity of texts, frequency of texts, duration of intervention, framework or model used, access to service, bidirectionality, content of texts, co-design or patient-oriented); (5) *demographics* (ie, person responding to texts, training of intervention deliverer, community characteristics, sex, race, average age, age range for inclusion in study, cultural relevance, reasons for low uptake/nonadherence, country); (6) *implementation* (ie, oversight, honorarium or credit, technology quality and performance, cost reported, security and privacy, interoperability, communication); and (7) *recommendations* (ie, recommendations for practice, policy, and research made by the primary study authors).

The form was piloted independently by two reviewers (Swati Rathore and SM) for 3 articles to determine if both parties were in agreement and to see if any clarifications were needed. This enabled an early check on consistency and relevance. Data extraction was then split between the two coders, and discrepancies or questions were resolved by consensus with input from another reviewer (LW).

### Analysis

All data for this scoping review were entered in Microsoft Excel (Microsoft Corporation). After data cleaning, a descriptive, analytical approach was used to generate summary statistics (counts, percentages, etc) of the data extracted for key sections 1-6. For section 7, key themes and issues from each study were identified by scrutinizing the results and discussion sections using thematic content analysis.

## Results

### Study Characteristics

**Figure 1:**
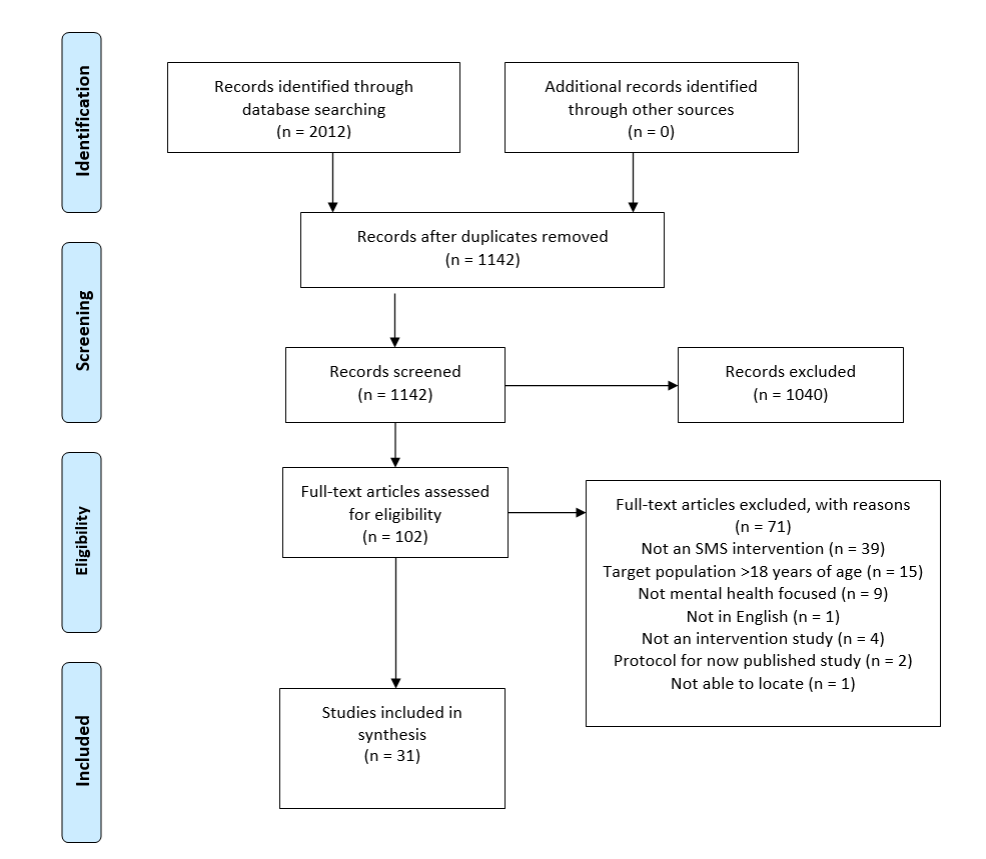
PRISMA flow diagram.

We screened 1142 abstracts for possible inclusion (Figure 1). After title and abstract screening, 102 full-text articles were screened for eligibility, with 71 articles excluded at this stage. Thirty-one studies were included, with 28 published in peer-review journals, 2 dissertations, and 1 abstract that included outcome data [48-78].

Included articles were published between 2013 and 2020. Of the 31 included studies, 18 (58%) were conducted in the United States. With respect to study design, 29% (9/31) incorporated randomization, 19% (6/31) were qualitative designs, and 52% (16/31) were observational or cross-sectional. In 68% (21/31) of the studies, fewer than 100 study participants were exposed to the intervention or service (see Multimedia Appendix 2).

### Measures and Outcomes

In additional to bespoke measures, over forty unique standardized outcome measures were used across the studies, measuring a wide range of symptom, global functioning, and therapeutic experience self-report scales and inventories. The measures categories with the widest use were intervention engagement (18/31; eg, number of texts sent, number of URL links clicked), cognitions (16/31; eg, disease knowledge, self-awareness), and acceptability (16/31; eg, satisfaction). Adherence to a predefined treatment protocol was measured in 7 studies, typically for substance use–related interventions (eg, reduction in number of drinks consumed). Fewer studies tracked social/relational outcomes (5/31; eg, quality of social relationships) or physical changes (2/31; eg, blood alcohol level). Forty-two percent of the studies (13/31) explicitly reported some evidence that the measures used were validated or reliable (eg, test-retest, internal consistency, and content validity checks). Twenty-six percent (8/31) reported outcomes that were measured immediately following exposure to the intervention. An additional 32% (10/31) measured outcomes within 3 months of intervention completion, but not immediately following, and 3 studies completed outcome tracking at 6 months postintervention or longer. Studies by Gonzales et al [59] and Whittaker et al [78] included a measure 12 months postintervention. Eighty-seven percent of studies (27/31) reported on at least one positive outcome including, for example, lowered stress [49], cost efficiency [52], less substance use [57], and treatment protocol adherence [51]. In the only randomized controlled trial of an intervention for adolescent depression that enrolled more than 500 participants, researchers found no evidence of benefit in depressive symptoms from a cognitive behavioral therapy–based SMS text message intervention compared to a control program [78]. However, youth reported finding the program helpful. One study reported not being able to complete outcome measurement due to major recruitment and retention issues during implementation [71]. Another study reported an adverse event/safety issue related to the intervention [71]. Across the 16 studies reporting on acceptability measures, the majority of adolescent participants (ie, >70%) in each case study reported being satisfied with the intervention.

### Intervention Characteristics

Interventions ranged considerably in length/duration and intensity (see Multimedia Appendix 3). The models or frameworks most frequently cited for guiding intervention development were social cognitive theory (5/31) and cognitive behavioral therapy (3/31), with the health belief model (1/31) and normalization process theory (1/31) also explicitly identified. Nine studies reported that the intervention had been co-designed with adolescents. Texting frequency ranged from several times per day for one depression-focused study [56] to once every two weeks or month for another depression-focused study [54]. Most frequently, interventions were delivered in less than 12 weeks, and adolescents received 1-3 messages per week over that time. Thirty-five percent (11/31) of studies specifically indicated the intervention was for substance use or problem drinking, and 32% (10/31) focused on adolescents with depression. Almost two-thirds of studies (20/31) had bidirectional texting, either automated bounce-back prompts (eg, [57]) or person-driven (eg, [65]). The 2020 study by Haug et al [65] incorporated multiple uses of automated text messages where messages were used to push weblinks, video clips, and pictures in some instances but also used to help assess and deliver individualized feedback through quizzes (eg, “reply to this SMS with ‘yes’ or ‘no’… are you meeting friends or going out today?”). Based on responses, a tailored but automated prompt would be sent (eg, “Hey Mike, great plan! Take a moment and imagine exactly how you could implement this plan…. Have a nice evening!”). Text messages across all studies were typically designed to convey empathy and encouragement (eg, “Stop… Breathe… And think about how you got through difficult times before. You got this!” [56]) and to support coping. The content and authenticity of the messages were cited by adolescent respondents as key elements of their engagement. As one respondent in the Duan et al study [58] reflected, “Don’t include messages that make me feel like my parents are teaching me something, I need encouragement from a friend, not from a teacher or parent”. Among studies utilizing bidirectional texting, 60% (12/20) required the researcher to respond to at least some of the texts. In 29% of studies (9/31), adolescents had the option to self-refer to the intervention. All other instances required provider or researcher referral or recruitment pathways. Two studies reported offering the intervention in more than one language.

### Implementation Contexts and Features

Community characteristics of adolescents enrolled in the interventions were explicitly described in only 10 of 31 studies and included limited details on community socioeconomic status, urban/rural geographic location, or generic statements related to culturally diverse community samples. Demographic information on the adolescent study participants revealed that most of the interventions were designed to target 13- to 17-year-olds. In 5 studies, the intended age range of end users covered more than 10 years (eg, Antiss and Davies, 2015 [49], ranged from 12 to 24 years of age). Forty-eight percent of the studies (15/31) reported providing adolescents with an honorarium or compensation for study participation. At least some level of training for the provider was noted in 35% (11/31) of studies. Twenty-nine percent of studies (9/31) reported on the technical quality or performance (eg, all messages were received). Sustainability (plans for scale-up or long-term operational planning) was discussed in 2 studies. Eight studies (26%) provided details on privacy and security of electronic data, which included things like requiring password-protected phones [61], limiting collection and storage of personal identifying health information [53], and confidentiality approval [74]. None of the studies reported on the direct costs of delivering the intervention. In 32% (10/31) of studies, the technical infrastructure used to deliver the text messages required some level of interoperability between interfaces (eg, text redirecting to a survey tool housed on another platform). As retention of adolescents in the studies was quite high, overall there was limited discussion of barriers to uptake or nonadherence. One author noted that even though only 38% of texts (eg, asking a participant to rate their mood from 1-10) were responded to by adolescents in the study, participants still reported improved outcomes [69].

### Practice, Policy, and Research Recommendations

Forty-two percent (13/31) of study authors reported that ease of use and broad accessibility were strengths of the services that made them feasible to roll out and resulted in high levels of uptake. Increased ability to tailor the scheduling of messages (eg, [67]; messages before or after school) or the content of messages (eg, [72]; including youth’s name in the texts) was noted by 35% (11/31) of authors as a way to practically improve interventions further. Multiple study authors noted the importance of working with youth to co-design and shape the messages. The intensity of exposure to texts (ie, time of day, number sent, and frequency) was pointed to as an important practical consideration by 19% (6/31) of authors. No studies explicitly addressed or identified specific policy-related implications of their findings. For example, there were no explicit recommendations around ethics of sending youth text messages, electronic communication policies, risk, privacy, or safety monitoring guidelines that would need to be in place for the service to operate beyond the research. Two primary sub-themes were identified in future research directions proposed by primary authors: (1) opportunities for comparative component studies that look at whether and which features (eg, tailoring, different messaging schedules, and different media) improve adherence or outcomes (15/31; 48%) and (2) need for well-powered randomized controlled trials (13/31; 42%).

## Discussion

### Principal Findings

To our knowledge, this review provides the first comprehensive mapping of the current literature on use of SMS text messaging for delivery of mental health and addiction interventions to adolescents. The aim was to detail outcomes being measured, clinical and technical features of interventions, implementation contexts in which they have been studied, and the ensuing recommendations made by primary authors to support innovative research in the field moving forward. Findings suggest a growing evidence base regarding text message interventions for adolescent mental health and addictions. These interventions appear to be highly acceptable and accessible to adolescents, easy to use, and have at least some positive impacts according to most studies. While these findings are consistent with the literature among adults receiving physical and mental health text message–delivered services [35], the research literature remains limited in several important ways. Overall, there are significant limitations in the trial designs. These include issues with small sample sizes (eg, pilot studies of less than 100 participants are typical), limited intervention duration, lack of randomized designs, nonstandard outcome measures and definitions, and few repeated measures in studies with longitudinal designs.

Limited grounding in, or at least reporting on, theoretical frameworks that drive intervention development was observed in this review. While most interventions tended to follow a basic architecture of less than 12 weeks and 2-3 messages per week, the content, levels of interactivity and personalization, use of media, and underpinning aims (eg, behavioral activation, acquisition of new knowledge, emotional regulation) were vastly different. Significant heterogeneity in intervention features was noted in our review, such as fixed message content or customized content; fixed-frequency or real-time support; standardized versus personalized messages; and unidirectional versus bidirectional communication. Without theoretical and therapeutic models to define and articulate how these intervention elements work to produce which outcomes (ie, satisfaction versus clinical change versus changes in thoughts and feelings), there is limited ability to generate and replicate rigorous hypothesis testing or clarify which mental health and addiction conditions at which level of illness severity might be best suited for SMS text messaging interventions. This finding further supports recommendations for standardized reporting of theories of change for behavioral interventions in academic research [79].

As this field emerges, high-quality studies will be required, despite rapidly evolving technical capabilities that can outdate services before they are able to be scaled up [80]. Preliminary usage data from primary studies in this review suggest adolescents will use and are satisfied with SMS text messaging services, but how satisfaction and engagement relate to direct health outcomes is unclear. In this review, over half of the included studies assessed some measure of acceptability or satisfaction. High scores on satisfaction outcomes across types of interventions, intensities, and countries of use point to possible ceiling effects of satisfaction measures [81]. Generating new knowledge about predictors of satisfaction and use (eg, treatment readiness, non–health-related texting use, degree of co-design [82]) could more usefully inform clinical practice guidelines for where these interventions are best matched to the needs of which adolescents, and under which circumstances. For example, adolescents across studies reported finding interventions helpful and acceptable and are satisfied with the experience even when they do not produce clinically significant changes (eg, [78]). The findings of this review have important implications for decision makers who are mandated to integrate services that show a return on investment. Mixed-effects studies that show interventions are “liked,” but have little impact on quality of life or symptoms, may present challenges for scale-up. Unpacking the relationship between perceived acceptability and clinical change will be an important direction for future research on SMS text messaging interventions. Better understanding of unidirectional versus bidirectional messaging design and their impact on engagement and exposure to the “dose” of SMS text messaging interventions could add needed insights. Preliminary research in other health fields with this population has established that bidirectional texting increases intervention effectiveness [83] and is a valuable line of inquiry for mental health and addiction–focused interventions.

Frequency and duration of interventions in this review, even for the same presenting clinical condition, ranged considerably from multiple times a day to several texts over the course of months. Future studies should consider the impact of habituation, response fatigue, and perceived “intrusiveness” of texts among adolescents using these interventions. Generating new knowledge about intervention intensity might also inform decision makers about where interventions “sit” within clinical workflow and provider/client relationships. For example, two-thirds of the studies in the review included some form of bidirectional interaction. It is possible that just messaging back and forth with clinic or research staff sufficiently evokes a relationship dynamic that bolsters perceived social connectedness and sense of well-being. Future work is needed to determine if it is the *presence* of ongoing communication at all or the specific therapeutic *content* of SMS text messaging interventions that results in improved outcomes. Studies in our review generally lacked details about how theory or therapeutic principles guided the content, frequency, and duration decisions around texts being sent to youth. It is promising that a number of studies reported some level of adolescent engagement as co-designers to potentially explicate some of these mechanisms of change and decisions around frequency and intensity. Richer descriptions of co-design models and theoretical frameworks used to shape intervention development would be beneficial.

This review points to methodological decisions and implementation considerations (eg, study remuneration practices, self-referral versus provider referral, and researcher-led communication) that might impact adherence and outcomes once interventions are rolled out beyond the research cycle into full-service delivery. These will be important for future researchers to consider and suggest that hybrid implementation/effectiveness studies could be a valuable study design for this field. For example, nearly half of the studies in the review reported providing remuneration for study participants. Would engagement be as high if youth did not receive remuneration for participating, but the service was offered as standard of care? Over a third of the studies reported that human resources required specialized training in order to support the SMS text messaging intervention or that technical troubleshooting for youth was required. If those tasks were to be integrated into a clinician’s existing workflow, how might that impact costs or sustainability? A significant gap identified in our review of implementation features was that none of the studies in the review provided costing information. The ability of this field to communicate cost-effectiveness data to policy makers that incorporate implementation costs is central to the likelihood of interventions being adopted by practitioners and would be in alignment with emerging standards for reporting [84]. Future studies could look to advances in implementation science to incorporate methodologies and outcomes that address these issues. In addition, cross-disciplinary work with fields like human factors engineering and health systems engineering could be particularly valuable for understanding how the integration of SMS text messaging interventions relates to workflow [85].

A systematic review of the use of research evidence in public health decision-making processes recommended that more research target the needs of decision makers [86]. A strength of this review is that in adopting Levac’s steps for scoping reviews, the research questions, data extraction and analysis, and reporting approaches were codeveloped with an interdisciplinary team of people with lived experience in mental health services and clinicians as well as local health system decision makers, through the MSSU. Their involvement was critical in identifying the diverse data of interest to be explored in this review. This scoping review, therefore, is set apart from other recent reviews by systematically highlighting persistent reporting gaps in the costing, sustainability planning, privacy and security, and technical infrastructure elements of SMS text messaging behavioral interventions for adolescents that are vital for translating research into real-world services. Knowing what has not been reported in the published literature is equally important for decision makers who are tasked with evaluating risks and return on investment. Recommendations for reporting on behavioral interventions have been around for over a decade [87,88] as well as new guidelines focused on mobile phone–based intervention reporting [89]. Gaps outlined in this review highlight knowledge-sharing opportunities on these important elements. Researchers in this rapidly advancing field can support decision makers to more quickly mobilize efforts to integrate effective, evidence-based SMS text messaging interventions by following these guidelines and integrating locally relevant information needs.

### Limitations

There are several limitations to this review. Although inclusion criteria were kept as broad as possible while maintaining focus on the research question, only 31 studies met inclusion criteria. While this is a significantly larger number than any of the other related recent reviews, a search of the grey literature was not completed. The search strategy was also limited as a result of search terminology related to mental health and addictions, which has an extremely diffused and nuanced lexicon across different fields. This issue has been raised by other researchers [90] and reported as a significant obstacle to integrating mental health services within broader health care systems [91]. This relatively small and highly heterogeneous sample raises an issue of publication bias and increases the importance of assessing for bias within individual studies. Within currently recommended scoping review methods, quality appraisals are not typically undertaken. While this review did not conduct risk of bias appraisals, systematically broad mapping of basic study methods did identify discrete areas of methodological weakness, which have been detailed and summarized to inform future work. In the context of this burgeoning intervention literature, it was important to include a range of designs to fully scope what is currently known. Finally, this review combined results from trials with early pilot studies, program evaluations, and qualitative investigations, resulting in a wide range of interventions, follow-up durations, and study aims. A review of only high-quality studies may produce different summary conclusions.

### Conclusion

Text message interventions are increasingly explored in adolescent mental health and addictions. However, the research to date has important limitations in terms of the heterogeneity of interventions and implementation characteristics of studies. There are significant gaps regarding the hypothesized theoretical and therapeutic mechanisms driving observed outcomes, which contributes to the lag in translating research into policy and practice. Impact on direct health outcomes, cost considerations, predictors of participant engagement, and ongoing sustainability or return on investment are underreported aspects of interventions studied to date. While studies broadly reported high levels of satisfaction with SMS text messaging interventions among adolescents, rigorous study designs are needed to parse out success features and how satisfaction related to engagement. Ideally, future research should formally compare characteristics (eg, number of texts, frequency, duration) in order to measure and adjust each of these parameters to meet the needs of adolescents as they change over time. Multiple lines of innovative inquiry in the use of SMS text messaging interventions for adolescent mental health and addictions are possible and promising.

## Appendix

Multimedia Appendix 1 Search strategy.

Multimedia Appendix 2 Characteristics of included studies.

Multimedia Appendix 3 Overview of intervention features and targeted population.

## Abbreviations

MSSU: Maritime SPOR SUPPORT Unit
PRISMA: Preferred Reporting Items for Systematic Reviews and Meta-Analyses
