# Supplementary material for: Text Message Interventions in Adolescent Mental Health and Addiction Services: Scoping Review
Source: JMIR Ment Health. 2021 Jan 8;8(1):e16508. doi: 10.2196/16508 (PMC7822725; doi:10.2196/16508)
Supplement: Multimedia Appendix 1 [file mental_v8i1e16508_app1.docx]

Appendix 1. Example search strategy

Ovid MEDLINE(R) and Epub Ahead of Print, In-Process & Other Non-Indexed Citations, Daily and Versions(R)

| 1 | Text Messaging/ |
| --- | --- |
| 2 | (short messag* service* or sms).ti,ab. |
| 3 | (text messag* or texting or texts).ti,ab. |
| 4 | or/1-3 |
| 5 | exp "Appointments and Schedules"/ |
| 6 | Reminder Systems/ |
| 7 | (active* adj wait*).ti,ab. |
| 8 | appointment*.ti,ab. |
| 9 | education*.ti,ab. |
| 10 | follow* up*.ti,ab. |
| 11 | outpatient*.ti,ab. |
| 12 | (pre intervention or post intervention).ti,ab. |
| 13 | (prompt* or remind*).ti,ab. |
| 14 | refer*.ti,ab. |
| 15 | schedul*.ti,ab. |
| 16 | support*.ti,ab. |
| 17 | (wait* list* or waitlist*).ti,ab. |
| 18 | or/5-17 |
| 19 | exp Anxiety/ |
| 20 | Depression/ |
| 21 | exp Mental Disorders/ |
| 22 | Mental Health/ |
| 23 | exp Mental Health Services/ |
| 24 | exp Suicide/ |
| 25 | ((affective or conduct or eating or mood or personality) adj disorder*).ti,ab. |
| 26 | (anorexi* or bulimi*).ti,ab. |
| 27 | (anxiet* or anxious).ti,ab. |
| 28 | bipolar.ti,ab. |
| 29 | counsel*.ti,ab. |
| 30 | depress*.ti,ab. |
| 31 | ((emotional or mental or psychological or social) adj2 (well being or wellbeing or wellness)).ti,ab. |
| 32 | ((mental* or psychiatric* or psychological*) adj2 (disease* or disorder* or health* or illness* or patient* or service*)).ti,ab. |
| 33 | (neurotic* or neuros#s).ti,ab. |
| 34 | panic*.ti,ab. |
| 35 | schizophreni*.ti,ab. |
| 36 | social work*.ti,ab. |
| 37 | stress*.ti,ab. |
| 38 | suicid*.ti,ab. |
| 39 | (trauma* or posttrauma* or ptsd).ti,ab. |
| 40 | addict*.ti,ab. |
| 41 | (alcohol* or drink*).ti,ab. |
| 42 | (amphetamine* or cannabis or cocaine or heroin or marijuana or meth or opioid* or pot or smok* or tobacco).ti,ab. |
| 43 | ((drug* or substance*) adj2 (abuse* or depend* or disorder* or habit* or "use" or user* or using)).ti,ab. |
| 44 | or/19-43 |
| 45 | Adolescent/ |
| 46 | (adolescen* or high school* or junior high or middle school* or secondary school* or teen* or youth*).ti,ab. |
| 47 | or/45-46 |
| 48 | 4 and 18 and 44 and 47 |
