# Supplementary material for: Text Message Interventions in Adolescent Mental Health and Addiction Services: Scoping Review
Source: JMIR Ment Health. 2021 Jan 8;8(1):e16508. doi: 10.2196/16508 (PMC7822725; doi:10.2196/16508)
Supplement: Multimedia Appendix 2 [file mental_v8i1e16508_app2.docx]

Multimedia Appendix 2. Characteristics of Included Studies

| **Author**  **(Yr)** | **Coun.** | **Name** | **Design** | **Sample** | **Measures** | **Follow-up freq.** | **Main Outcomes** |
| --- | --- | --- | --- | --- | --- | --- | --- |
| Ammerman  (2015)[48] | US | Text messaging health intervention | Pilot | <100 | N/A | Immediate: after completing intervention | Subjective improvement in health behavior, secondary to information and advice received by text. |
| Anstiss  (2015)[49] | NZ | Reach Out, Rise Up | Pre-post | <100 | GAD-7; PHQ-9 | Not reported | Post intervention anxiety and depression scores were significantly lower than their pre-intervention scores. |
| Bjørnholt  (2016)[50] | DK | Text messaging for medical compliance | Randomized | <100 | READ; TEC; MFQ; DES-T; ADHD-RS | Not immediate: within 3 months of completing | Compliance was not significantly associated with the text message intervention in any of the drug interventions |
| Bopp  (2015)[51] | US | True Colours | Randomized | <100 | QIDS-SR; K-SADS; ASRM | Immediate: after completing intervention | Control participants were significantly more adherent to the weekly protocol than bipolar participants. |
| Branson  (2013)[52] | US | Text messaging reminders | Randomized | <100 | GAIN-SS | Not immediate: within 3 months of completing | The patient satisfaction measure was significant. |
| Chandra  (2014)[53] | IN | SMS for mental health | Qualitative | <100 | N/A | Not immediate: within 3 months of completing | Majority of participants called back, asking for mental health services, and felt supported with messages. |
| Chen  (2017)[54] | US | Text message intervention for adolescent depression | Pre-post | <100 | PHQ-9 | Immediate: after completing intervention | 100% weekly response rate for mood, sleep, and PHQ-9. No correlation between mood, sleep, and PHQ9. Patient reported satisfaction. |
| Connolly  (2017)[55] | US | EMA design | Pre-post | 101-500 | SADS-L, SRRS, RRS, SRR-S, MRSI-A, SDS | Immediate: after completing intervention | Momentary ruminative self-focus did not independently predict an increase in depressive symptoms at the next alert. |
| Czyz (2020)[56] | US | Text messaging intervention development | Pilot | <100 | 5-point scale to rate messages, daily surveys, open-ended feedback | Not immediate, but within 3 months | Participants wanted option to request an additional text message each day and stop messages |
| Dennis  (2015)[57] | US | Feasibility of using the smartphone to provide recovery support | Qualitative | <100 | GAIN-Q3 | Immediate: after completing intervention | Both the unrecognized risk group (50%), and current use (96%) reported significant. |
| Duan (2020)[58] | CN | Text messaging intervention development | Qualitative | <100 | N/A | N/A | Participants were receptive to receiving messages, short frequency and duration of messages, caring content in messages, specific timing for message delivery |
| Gonzales, Anglin  (2014)[59] | US | Project ESQYIR | Randomized | <100 | T-ASI; GAIN; BAM | Not immediate: within 3 months of completing | Intervention group was significantly less likely to relapse to primary care, less substance abuse problems or severity. |
| Gonzales, Anglin  (2014) [60] | US | Substance abuse treatment program | Qualitative | <100 | N/A | N/A | 70% of youth positively endorsed text messaging, |
| Gonzales et al.  (2016)[61] | US | Project ESQYIR (post-participation) | Randomized | <100 | BAM | 1. not immediate but within 3 months  2. 4<= 6 months  3. 7<= 12 months  4. >12 months | Test positive for primary drug compared to youth in ‘aftercare as usual’ condition Significantly higher self-efficacy/confidence to abstain during recovery |
| Haug  (2013)[62] | CH | Alk-Check | Pre-post | 101-500 | DDQ; Tobacco Smoking; Physical activity | Not immediate: within 3 months of completing | Decreases in the percentage of persons with RSOD, alcohol related problems, and in the mean number of standard drinks per week. |
| Haug, Paz Castro, Kowatsch  (2017)[63] | CH | MobileCoach Alcohol | Randomized | 501+ | Prevalence, frequency, quantity of alcohol consumption; peak blood alcohol concentration; AUDIT | 4 <=6 months | RSOD prevalence decreased by 5.9% in the intervention group and increased by 2.6% in the control group. No significant group differences were observed for most secondary outcomes: |
| Haug, Paz Castro, Meyer  (2017)[64] | CH | Ready4life | Pre-post | 501+ | PSS; SSKJ 3-8; Assertion inventory (social skills) | 4 <=6 months | Decreased perceived stress and increases in several life skills. The proportion of adolescents with at-risk alcohol use declined. No significant changes for tobacco or cannabis use. |
| Haug (2020)[65] | CH | MobileCoach Alcohol | Randomized Controlled Crossover Trial | 100-500 | AUDIT-C | N/A | Reduction in number of alcoholic drinks with friends or when going out by one standard drink |
| Hickman  (2018)[66] | US | Educational text-message and URL intervention | Program Evaluation | 101-500 | Usage measures: uptake, check ins | N/A | Adolescents learned something new, made a behavioral change, and overall liked the text messages |
| Hospital  (2016)[67] | US | Developing an SMS intervention for preventing underage drinking among Hispanic teens | Qualitative | <100 | N/A | N/A | Design recommendations: 2 txts /week; send at high-risk times; not bi-directional;  positive sounding; focus on knowledge, self-efficacy, social support, offered in multiple languages |
| Hu  (2018)[68] | US | Project READY | Unclear | <100 | Customary Drinking and Drug Use Record;  What I Got from Treatment;  Self-efficacy | Immediate: after completing intervention (Follow-up at Week 4)  Within 3 months of completing (Second follow-up at Week 8) | Text coaching predicted greater reduction in alcohol use at end of treatment but not marijuana use.. |
| Kobak  (2015)[69] | US | Technology Enhanced Cognitive Behavioural Therapy | Pilot | <100 | Knowledge; SUS; QIDS-A-Pat; CGI-I; CGI-S; TASA | N/A | 95% of teens said reviewing their text messages with their therapist was helpful, and all said they would use text messaging in treatment again. A significant reduction in depression. |
| McKnight  (2017)[70] | UK | True Colours | Cohort | 101-500 | QIDS; ASRM | Unclear | Compliance with weekly questionnaires was generally high (median, 92% of weeks). No significant differences in mood symptoms or variability were observed between bipolar I and II patients |
| Owens  (2016)[71] | UK | TeenTEXT | Unclear | <100 | Not reported due to recruitment issues. | N/A | Barriers to implementation: heavy workloads and high stress levels; organizational gatekeeping practices, perceived burdensomeness and technophobia on the part of clinicians. |
| Pisani  (2018)[72] | US | Text4Strength | Pilot | <100 | SMFQ; K10; GAD-7; DERS; Usage (interactions with texts, videos); Appeal;  Perceived benefit | Not immediate: within 3 months of completing | 91% of youth responded to at least one sequence. With the exception of spirituality and generosity topics a moderate number of student replies. Students at different levels of distress interacted with the sequences at similar rates. 70% found the texts useful and 90% thought that the intervention should be repeated. |
| Pisani (2019)[73] | US | Educating Peer Leaders to Prevent Substance Use | Pilot | <100 | Parent acceptability, peer leader surveys and participation, interaction with texts | N/A | The texting intervention was well-received by peer leaders and associated with participation in school-based activities |
| Ranney  (2014)[74] | US | Patient intervention development | Qualitative | <100 | N/A | N/A | Design recommendations: Peer endorsement may increase uptake. Messages should be simple and positive. Tone should be conversational but not slang filled. Messages may be automated but must be individually tailored.. Multimedia may be helpful but is not necessary. |
| Ranney  (2018)[75] | US | iDOVE | Pilot RCT | <100 | BDI-2; Revised CTS-2); CSQ-8; CTRS and MITI | Immediate: after completing intervention (Follow-up at Week 8)  Within 3 months of completing (Second follow-up at Week 16) | 95% and 91% completing 8-week and 16-week follow-ups, respectively. Improved depressive symptoms and physical peer violence among the more symptomatic youth in the intervention group |
| Sindahl (2019)[76] | DK | BorneTelefonen | Retrospective Observational Study | 100-500 | ES-Q, FU-Q | Immediate: after completing intervention (and again at 2 weeks) | 35.9% of suicidal children reported feeling better immediately following the session and over half ended the session with a plan of action. 23.9% felt better at 2-week follow up but 37% reported feeling worse. |
| Summerhurst  (2018)[77] ] | CA | FEMAP | Evaluation | 101-500 | N/A | N/A | Primarily used for the scheduling of appointments (58.9%) |
| Whittaker (2017)[78] | NZ | MEMO CBT | Double blind randomized placebo-controlled trial | 501+ | CDRS-R, RAS-2, PQ-LES-Q, MFQ, YRBS, K-SADS, post-intervention satisfaction questionnaire | 7<= 12 months | There was no association between the mobile phone CBT intervention and control program in relation to preventing depression |

**General Anxiety Disorder scale (GAD-7), Patient Health Questionnaire (PHQ-9); Resilience Scale for Adolescents (READ), Traumatic Experiences Checklist (TEC), Mood and Feelings Questionnaire (MFQ), Dissociative Experiences Scale-Taxon (DES-T), ADHD - Rating Scale (ADHD-RS); True Colours Self-Management System (TCSMS), Quick Inventory of Depressive Symptoms - Self-Report QIDS-SR), Altman Self-Rating Mania Scale (ASRM), Kiddie Schedule for Affective Disorders Depression and Mania Rating Scales (K-SADS); Global Appraisal of Individual Needs – Short Screener (GAIN-SS); Short Message Service (SMS); Schedule of Affective Disorders and Schizophrenia, Lifetime (SADS-L), Stress-Reactive Rumination Scale (SRRS), Ruminative Response Scale of the Response Styles Questionnaire (RRS), Beck Depression Inventory (BDI-II), Stress-Reactive Rumination Scale-State (SRRS-S), Momentary Ruminative Self-Focus Inventory-Abbreviated (MRSI-A); Global Appraisal of Individual Needs – Quick Version 3 (GAIN-Q3), Educating & Supporting Inquisitive Youth in Recovery (ESQYIR), Teen-Addiction Severity Index (T-ASI), Global Appraisal Inventory of Needs (GAIN), Brief Addiction Monitor (BAM); Risky single-occasion drinking (RSOD), Daily Drinking Questionnaire (DDQ); Perceived Stress Scale (PSS), Questionnaire for the Measurement of Stress and Coping in Children and Adolescents (SSKJ 3-8); Alcohol Use Disorders Identification Test Concise (AUDIT-C); System Usability Scale (SUS), Quick Inventory of Depressive Symptomatology–Adolescent Version (QIDS-A-Pat), Clinician Global Ratings of Improvement (CGI-I), Clinician Global Ratings of Severity (CGI-S), Therapeutic Alliance Scale for Adolescents (TASA); Quick Inventory of Depression Symptomatology (QIDS), ASRM (Altman Self-Rating Mania Scale); intervention for Depression and Violence prevention in the Emergency department (iDOVE); Short Mood and Feelings Questionnaire (SMFQ); Kessler Psychological Distress Scale (K10); Difficulties in Emotion Regulation Scale (DERS); Beck Depression Inventory-2 (BDI-2), Conflict Tactics Scale-2 (CTS-2), Cognitive Therapy Rating Scale (CTRS), Client Satisfaction Questionnaire-8 (CSQ-8),Motivational Interviewing Therapy Integrity (MITI); End Session Questionnaire (ES-Q), Follow Up Questionnaire (FU-Q); First Episode Mood and Anxiety Program (FEMAP); Child Depression Rating Scale-Revised (CDRS-R), Reynold’s Adolescent Depression Rating Scale – 2nd Edition (RADS-2), Pediatric Quality of Life Enjoyment and Satisfaction Questionnaire (PQ-LES-Q), Youth Risk Behaviour Survey (YRBS), Kiddie-Schedule for Affective Disorders and Schizophrenia (K-SADS); United States of America (US); Switzerland (CH); Canada (CA); United Kingdom (UK); Denmark (DK); New Zealand (NZ); India (IN); China (CN).*
