# Supplementary material for: Text Message Interventions in Adolescent Mental Health and Addiction Services: Scoping Review
Source: JMIR Ment Health. 2021 Jan 8;8(1):e16508. doi: 10.2196/16508 (PMC7822725; doi:10.2196/16508)
Supplement: Multimedia Appendix 3 [file mental_v8i1e16508_app3.docx]

Multimedia Appendix 3. *Overview of intervention features and targeted population*

| **Author**  **(Yr)** | **Popul.** | **Dur.** | **Freq.** | **Age** | **Access** | **Bi-direct. (Y/N)** | **Responder** | **Supervisor** |
| --- | --- | --- | --- | --- | --- | --- | --- | --- |
| Ammerman  (2015) | At-risk youth | 14 wks | 3/wk | Unclear | R | Y | C | R |
| Anstiss  (2015) | Anxiety and/or depression | 10 wks | 3/wk | 12-24 | R | N | N/A | R |
| Bjørnholt  (2016) | Vulnerable youth with psychiatric disorders | Until end of treatment | 7/wk (1/day) | 15-20 | C | N | N/A | C |
| Bopp  (2015) | Bipolar disorder | 36 wks | 1/wk | 13-18 | S+R | Y | R | R |
| Branson  (2013) | Outpatient mental health patients | 3 mos | 1/wk | 13-17 | S+C | N | N/A | R+C |
| Chandra  (2014) | Female youth in urban slums | 1 mo | 7/wk (1/day) | 16-18 | Unclear | Y | C+R | R |
| Chen  (2017) | Depression | 2 to 4 mos | 1/2 wks or month | Unclear | C | Y | R | R |
| Connolly  (2017) | Depression | Unclear | 28/wk (4/day) | Unclear | R | Y | R | R |
| Czyz  (2020) | Adolescents at elevated suicide risk | 4 wks | 2/day | 13-17 | C | N/A | N/A | R |
| Dennis  (2015) | Substance abuse disorder | 6 wks | 42/wk (6/day) | 14-18 | R | Y | R | R |
| Duan (2020) | Adolescents who self-harm | N/A | N/A | 12-17 | C | N/A | N/A | R |
| Gonzales, Ang  (2014) | Substance abuse disorder | 12 wks | Unclear | 14-26 | R | Y | Auto | R |
| Gonzales, Anglin  (2014) | Substance abuse disorder | 2 hrs | Unclear | 12-24 | R | N/A | N/A | R |
| Gonzales  (2016) | Substance abuse disorder | 12 wks | 7/week (1/day) | 12-17 | R | Y | R | R |
| Haug  (2013) | Problem drinking | 12 wks | 1-2/wk | 16-20 | R | N | N/A | R |
| Haug, Paz Castro, Kowatsch  (2017) | Problem drinking | 3 mos | 1-2/wk | 16-19 | R | N | N/A | R |
| Haug, Paz Castro, Meyer  (2017) | Substance use (prevention) | 6 mos | 2-4/wk | 16-19 | R | Y | N/A | R |
| Haug (2020) | Adolescents who have recently binge drank | 12 wks | 3/week | 16.1 avg | R | Y | Auto | R |
| Hickman  (2018) | Healthy behavior promotion/ risky behavior prevention | 12 wks | 1/wk | 14-18 | R | Y | Unclear | R |
| Hospital  (2016) | Problem drinking | Unclear | Unclear | 14-19 | S+R | N | N/A | R |
| Hu  (2018) | Substance use | 4 wks | Multiple texts 2 X/wk | 14-19 | S+P | Y | C | C |
| Kobak  (2015) | Depression | 12 wks | Not clear | 12-17 | C | Y | C | R |
| McKnight  (2017) | Bipolar disorder | Up to 81 mos | Varied | 16-76 | S+C | Y | R | R |
| Owens  (2016) | Self-harm | 6 mos | Not completed | 12-18 | C | Y | C | R |
| Pisani  (2018) | Suicide (prevention) | 9 wks | Varied | 13-15 | R | Y | R | R |
| Pisani (2019) | Nominated peer leaders to prevent substance use | 4 months | 2-3/week | 13.8 avg | P/T | Y | Auto | R |
| Ranney  (2014) | Violence and depression | Unclear | Unclear | 13-17 | R | N | N/A | C+R |
| Ranney  (2018) | Violence and depression (prevention) | 8 wks | 7/ wk (1/day) | 13-17 | R | Y | R | C+R |
| Sindahl (2019) | Youth accessing helpline for suicide ideation | As needed sessions | N/A | <23 | S | Y | C | Unclear |
| Summerhurst  (2018) | Mood and anxiety | Up to 2 yrs | Unclear | 16-25 | C | Y | C+R | C+R |
| Whittaker (2017) | High school students at risk of depression | 9 weeks | 2/day | 13-17 | R | N | N/A | R |

*C= Clinician/practitioner/counsellor/therapist*

*N/A = Not Applicable*

*R= Researcher*

*S= Self*

*P= Peer*

*T = Teacher*
